# Advancing gender equity in primary health care: Lessons from integrating gender-responsive strategies into service delivery in Ethiopia

**DOI:** 10.1371/journal.pgph.0006764

**Published:** 2026-07-16

**Authors:** Gizachew Tadele Tiruneh, Agumasie Semahegn, Biruhtesfa Bekele Shiferaw, Mesele Damte Argaw, Nebreed Fesseha, Chala Tesfaye, Temesgen Ayehu, Biruk Bogale, Metadel Tesfaye, Addis Girma, Wuleta Betemariam, Alex Munive, Frank DelPizzo, Lidiya Tefera, Helina Worku, Misrak Makonnen, Addis Tamire, Dessalew Emaway

**Affiliations:** 1 Improve Primary Health Care Service Delivery Project, JSI, Addis Ababa, Ethiopia; 2 Improve Primary Health Care Service Delivery Project, Amref Health Africa in Ethiopia, Addis Ababa, Ethiopia; 3 Reproductive, Maternal, Child, and Newborn Health Practice, SI, Washington D.C, United States of America; 4 Global Center for Gender Equality, Washington, D.C, United States of America; 5 Ethiopia Country Office, Gates Foundation, Addis Ababa, Ethiopia; School of Public Health, College of Health Science, Addis Ababa University, ETHIOPIA

## Abstract

Gender inequities remain a major barrier to women’s access to reproductive, maternal, newborn, and child health (RMNCH) services. To address this gap, the *Improve Primary Health Care Service Delivery (IPHCSD)* project integrated a set of gender-responsive strategies into primary health care (PHC) systems. This paper presents lessons from implementing gender-responsive strategies that empowered women and communities while enhancing access to essential health services. A quasi-experimental embedded implementation research with a participatory mixed-methods approach was employed. The study combined controlled before-and-after household studies, phenomenological research, gender analysis, and stakeholder engagement to identify gender-related barriers and co-design contextually appropriate interventions. A mixed-effect logistic regression model was fitted to estimate the net effect using a difference-in-difference (DiD) approach. The study identified substantial gender-related barriers to RMNCH service utilization, including restrictive social norms, high workloads for women, limited decision-making power and control over resources, and inadequate male involvement. In response, the project implemented a comprehensive gender integration strategy. This included strengthening women’s participation through learning and action groups, establishing gender-sensitive service standards, and expanded home visits and targeted outreach and promoting male engagement. These strategies demonstrated strong transformative potential of enhancing women’s agency and improving RMNCH service delivery and utilization. Quantitatively, the intervention significantly improved women’s agency in agrarian settings (DiD: + 13.6 percentage points; p < 0.001). Uptake of maternal and newborn health services also increased significantly: DiD of +15.8 percentage points (p < 0.001) and +12.3 percentage points (p = 0.031) under the community-based MNH delivery strategy, and +29.0 (agrarian) and +15.9 (pastoral) under the PLA strategy. Gender-integrated approaches strengthened women’s agency, improved access to and use of RMNCH services, and contributed to universal health coverage. Integrating gender considerations and enhancing men and youth involvement into PHC is essential for reducing disparities and building more inclusive health systems in vulnerable communities.

## Introduction

Although a high-quality primary health care (PHC) system can address most of the community health needs as the first point of service delivery, Ethiopia continues to experience high maternal and newborn mortality, with approximately 267 maternal deaths per 100,000 live births and 27 neonatal deaths per 1,000 live births [1,2], alongside substantial regional disparities that disproportionately affect rural and pastoralist regions such as Afar, Somali, and parts of Oromia and Amhara [3,4]. Gender inequities further undermine women’s and girls’ access to essential health services and remain a key driver of poor reproductive, maternal, newborn, and child health (RMNCH) outcomes [5–8]. On the demand side, factors such as gender-based divisions of labor, limited access to and control over resources, restrictive gender norms, lack of autonomy, and inadequate decision-making power, combined with supply-side challenges like systemic discrimination, and disrespectful or non-inclusive care practices, hinder women’s ability to access equitable and quality RMNCH services [9–11].

The international health agencies underscore the importance of addressing gender dynamics and promoting gender equality to improve RMNCH outcomes [12]. Gender integration refers to the systematic consideration of gender norms, roles, relations, and power dynamics throughout the program cycle and is commonly conceptualized along a continuum ranging from gender-blind and gender-exploitative approaches to gender-accommodating and gender-transformative approaches, with the latter seeking to address and transform inequitable gender norms and power relations [9,12,13]. Integrating gender into PHC approaches—including service design, delivery, quality improvement, community engagement, and monitoring and evaluation —can improve responsiveness, equity, and quality of care while enhancing women's agency and access to essential RMNCH services.—. Although integrating gender considerations throughout planning, design, implementation, and evaluation has been shown to improve intervention outcomes and promote equitable, high-quality care [9,12], gender integration remains inconsistent across project design, implementation, and evaluation, particularly within routine PHC systems [12].

The Government of Ethiopia has adopted the PHC approach over the past half-century, with further revitalization in the last two decades through the Health Extension Program (HEP) to ensure access to essential health services [14]. The Ministry of Health (MOH) has also prioritized gender mainstreaming in its strategic plan as a key approach to improving health care access for women and other vulnerable populations across the health system [15]. However, the systematic integration of gender considerations into RMNCH programs remains limited, resulting in persistently low health care service uptake among women and girls within the PHC system [14].

Since 2022, the *Improve Primary Health Care Service Delivery (IPHCSD)* project has been strengthening the country’s PHC system and enhancing RMNCH outcomes. The project has adopted a gender-responsive approach, focusing on identifying and addressing gender-related barriers that impact the demand for and utilization of PHC services. It examined gender-related barriers to accessing and utilizing PHC services, identified opportunities for embedding gender within the PHC framework, outlined the development of gender integration strategies and frameworks, and highlighted the impact of these efforts in empowering women and communities to enhance the uptake of RMNCH services [16]. This paper documented and synthesized lessons learned from the integration of gender-responsive approaches into the PHC service delivery system in the agrarian and pastoral contexts of Ethiopia, highlighting the design process, implementation strategies, and implications for improving equitable RMNCH service delivery.

## Methods

### Ethics statement

All primary data were collected after obtaining both verbal and written informed consent from each study participant. The study received ethical approval from the Institutional Review Board (IRB) of the Ethiopian Public Health Association (Ref. #: EPHA/OG/728/23, July 17, 2023; renewed Ref. #: EPHA/OG/466/24, August 07, 2024) for the household and qualitative surveys, and from the IRB of the Ethiopian Society of Sociologists, Social Workers, and Anthropologists (Ref. #: ESSSWA/L/AA/0452/2023) for the gender analysis.

Informed consent was obtained from all participants after providing comprehensive information about the study, including its purpose, potential benefits, risks, and their right to decline participation or skip any questions. For participants younger than 18 years of age, consent was obtained from their parents or legal guardians. Upon agreement to participate, the interviewer documented consent by marking the questionnaire and providing a digital signature below the consent statement. Interviews were conducted only after consent had been appropriately obtained and recorded.

All information collected from participants was treated with strict confidentiality. Data were de-identified prior to analysis, and appropriate safeguards were implemented to protect participants’ privacy and maintain the integrity of the study. The study was conducted in full compliance with the principles of the Declaration of Helsinki.

### Settings

The Ethiopian health system is organized into three levels of care: primary, secondary, and tertiary. The primary level includes health posts, health centers, and primary hospitals. In 2020, the MOH launched the HEP Optimization Roadmap (2020–2035) to enhance access to and the quality of essential health services at the PHC level [17]. The key strategic objectives of the roadmap focus on enhancing the quality, accessibility, and efficiency of PHC services by strengthening human resources, improving facility infrastructure, boosting community engagement, and integrating health services to address local health needs better. Community engagement strategies, such as Village Health Leaders (VHLs) and Women Development Armies, involve local volunteers who are recruited and trained to deliver basic health care services and promote health education in rural communities [17].

The IPHCSD project, has been implemented since April 2022 through a partnership between Amref Health Africa in Ethiopia and JSI, aims to strengthen the functionality and bidirectional linkages across PHC delivery platforms—health posts, health centers, and primary hospitals—to improve RMNCH outcomes. The project focuses on enhancing equitable access, service quality, technical oversight, accountability, and generating evidence to optimize PHC service delivery. Using the Networks of Care (NoCs) approach interconnects health facilities and health workers to foster relational linkages, improve care coordination, strengthen referral systems, and ultimately enhance the delivery of RMNCH services and outcomes at the woreda level [16,18,19]. The project has been designed to incorporate a gender-intentional approach as a minimum standard to achieve gender-transformative and inclusive results. As such, gender integration has been a central element of the project.

### Design

A multi-method embedded implementation research approach was employed for this project, including mapping the pathways for gender integration, comprehensive empathy-driven formative research, continuous engagement of key stakeholders, participatory co-design, implementation and evaluation techniques.

### Pathways to gender integration

**Fig 1:**
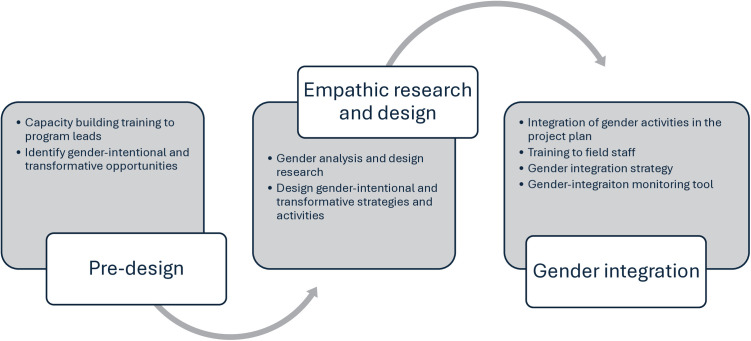
Gender integration in the primary health care system design and implementation pathways.

The project’s gender integration process was a systematic, evidence-based, and participatory pathway grounded in established gender and implementation science frameworks, including World health Organization gender mainstreaming in health systems [20], the United States Agency for International Development (USAID) Gender Integration Continuum [13], and the EngenderHealth Gender Integration Framework [21], alongside human-centered design principles aimed at embedding gender equity throughout its design and implementation strategies (Fig 1). During the pre-design phase, capacity-building training was provided to program leads to enhance their understanding of gender dynamics in seeking and accessing health services and integration practices. This phase also focused on identifying gender-intentional and transformative opportunities that could be leveraged to address existing gender-related systemic barriers in the health system and beneficiaries and promote equitable health and social outcomes.

In the empathic research and design phase, the project conducted a gender analysis and formative exploratory research to uncover the unique needs, challenges, and opportunities for women, girls, and vulnerable groups. Insights from this research informed the development of gender-intentional and transformative strategies and activities, ensuring that the project addressed the root-causes of gender disparities in health care access and quality.

The gender integration phase involved incorporating gender-focused activities into the overall project plan and providing training to field staff to equip them with the skills to implement these activities effectively. A comprehensive gender integration strategy was also developed to guide the project in promoting gender equity across all levels of implementation, ensuring that gender considerations were seamlessly embedded into the project's core strategies and activities.

### Empathic formative research

The project employed a comprehensive gender analysis between 24/08/2023 and 09/11/2023 to identify critical gender gaps, as well as demand-side and supply-side barriers affecting access to PHC services [22], followed by a household survey among women to assess women’s autonomy and attitudes toward gender equality. Using an empathic formative research approach, the study sought to deeply understand these barriers and identify opportunities within the PHC system. A combination of methodologies was utilized, including household surveys to gather quantitative data on health-seeking behaviors, programmatic qualitative studies to explore perceptions and challenges, and gender analysis to examine disparities and dynamics influencing service access. Human-centered design approaches enabled the co-creation of practical and culturally relevant solutions with stakeholders in both agrarian and pastoral settings. Collectively, these efforts laid the foundation for designing inclusive, gender-responsive, and context-tailored interventions.

Additional information was gathered during co-design sessions for the implementation strategy and consultative workshops. Furthermore, regular monitoring and evaluation data on the community-based delivery of RMNCH services and participatory and learning action (PLA) strategies were collected through routine project monitoring systems.

The formative household survey was conducted among women aged 15–49 years focusing on gender-related metrics such as decision-making power, male partner engagement, and access to resources. This was complemented by a concurrent phenomenological study, which included 34 in-depth interviews (IDIs), 35 key informant interviews (KIIs), and 12 focus group discussions (FGDs) with postpartum women, community leaders, program managers, and service providers to explore gender-related barriers [23]. An exploratory qualitative gender analysis was conducted between 24/08/2023 and 30/09/2023, involving 87 in-depth interviews and 134 discussants across 18 FGDs, to examine gender barriers in relation to the project’s primary focus areas [22]. Additionally, human-centered design techniques were used to engage 30 women, 10 husbands, and 10 service providers, uncovering insights and opportunities for gender integration (Table 1).

**Table 1:** Matrix of the formative research design, samples interviewed, focus areas, and key findings.

Research type	Samples interviewed	Focus areas	Key findings
Formative household survey	Women with 0–11 months children	Gender-related barriers to access to and use of RMNCH services examined	Low women’s autonomy in care-seeking, low partner involvement, and persistent gender norms limiting timely use of antenatal care, delivery, and PNC services more pronounced in pastoralist settings [23]
Programmatic qualitative study	12 FGDs, 34 IDIs and 35 KIIs	Concurrently with the household survey, gender-related barriers in-depth explored	Poor service readiness, missed opportunities for integrated care, and suboptimal client profiling and continuity of care; providers reported cognitive overload and limited supportive supervision [22,23]
Gender analysis	87 KIIs and 18 FGDs (136 participants)	Gender barriers across the access to quality PHC, and accountability system explored	Strong influence of patriarchal norms, low male engagement (about one-third accompaniment to antenatal care), acceptance of harmful norms (including justification of wife beating in some settings), and weak accountability mechanisms for gender equity in PHC [22]
Human-centered design	30 women, 10 husbands, and 10 service providers	Women’s insights on gender and opportunities for integration explored	Preference for respectful, client-centered care; need for privacy, flexible service hours, integrated RMNCH services, and simplified care pathways linking community and facility systems

### Participatory co-design and stakeholder engagement

The co-design of integrated gender-responsive intervention implementation strategy and its validation workshop with stakeholders and partners was conducted to corroborate the findings and develop actionable plans on five selected woredas from November 2023 to February 2024. A total of 254 participants (162 male and 92 female), representing diverse stakeholders, including the program managers, health care providers, and community representatives at each level of the health system and gender focal persons attended the validation workshop. During this collaborative workshop, participants recognized the resonance of the findings with their local contexts and collectively formulated an action plan that included improving responsive and equitable access through tailored service delivery and participatory women’s group learning and action, operationalization of gender-responsive PHC standards and development of gender-intentional tools and guidelines, and strengthening accountability through regular supportive supervision and gender-responsive monitoring and evaluation systems with defined indicators. This action plan served as a vital input during the subsequent development of the gender integration strategy.

### Data and data analysis

For this paper, data were drawn from multiple sources relevant to the design, implementation, and evaluation of the integrated gender-responsive PHC strategy. We analyzed before–after controlled household survey datasets, reviewed formative survey reports, and examined routine project monitoring data. The routine monitoring data consisted of longitudinal quantitative information collected by VHLs from enrolled women between 01/12/2023 and 30/06/2025 through home visits, starting in early pregnancy and continuing through the immediate postpartum period. At each visit, VHLs updated individual records, capturing data on the uptake of life-saving maternal and newborn health (MNH) interventions, medications, and maternal conditions during the intrapartum and postpartum phases, which were subsequently uploaded into a customized District Health Information Software 2 (DHIS2) Tracker tool.

The formative household survey included 2,617 women—786 from MNH intervention areas, 900 from PLA areas, and 931 from comparison areas—of whom 1,242 were from agrarian settings and 1,375 from pastoral settings. Similarly, at end-line (15/07/2025-10/09/2025), we conducted a follow-up household survey among 3,381 women: 1,071 from PLA areas, 852 from MNH areas, and 1,457 from comparison areas. Of these, 1,605 were from agrarian settings and 1,776 from pastoral settings. The surveys focused on gender-related indicators, including women’s decision-making power, male partner engagement, and access to resources.

A mixed-effects regression mode was fitted to estimate the difference-in-difference (DiD) to determine the effect of integrated gender-responsive approach to strengthen women’s agency and uptake of institutional delivery. The women’s agency for maternal and newborn health service index is a composite measure derived from 22 survey items across four domains: gender-equitable attitudes, decision-making power, control over financial resources, and mobility and access constraints. All items were recoded so that higher values consistently reflected greater agency. Principal component analysis was applied to construct the composite index, conducted separately for agrarian and pastoral settings to account for contextual differences in response patterns. The first principal component was retained as the latent agency score and standardized (z-score) within each context. For interpretability, the standardized score was rescaled to a 0–100 range using min–max normalization, with higher values indicating greater agency.

## Results

This section highlights the gender-related barriers to RMNCH service access identified through empathic research, the co-designed strategies and activities developed to address these barriers, the implementation strategies and gender integration frameworks established to incorporate gender into the PHC system effectively, and the impact of gender integration on empowering women and communities to improve the uptake of RMNCH services.

### Gender-related barriers to essential health services

Gender-related barriers to accessing, providing, and utilizing RMNCH services were deeply rooted in individual, interpersonal, community, and system-related factors. These barriers significantly hinder equitable access to care, particularly for women in rural areas, unmarried adolescents, and pregnant women constrained by restrictive cultural norms and beliefs. Factors such as heavy workloads, unequal division of labor, limited partner or family support, restricted autonomy, and lack of control over resources further limit women’s ability to seek timely care.

The project highlighted low levels of male involvement in maternal health, such as accompanying their wives to antenatal care clinics, especially in pastoral communities. The quantitative data revealed significant regional variations, further supporting the observed differences in RMNCH practices and attitudes across communities. Compounding these challenges was the absence of context-sensitive health services, insufficient supplies, and high opportunity costs—including lost time and income—when seeking care, which disproportionately impact vulnerable groups.

**Fig 2:**
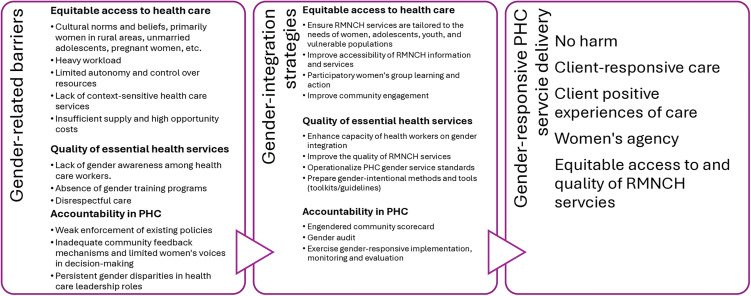
Gender-related barriers and strategies to address those barriers.

The quality of RMNCH services was further compromised by the lack of gender awareness among health care workers, the absence of gender-sensitive training programs, and the prevalence of disrespectful and suboptimal care. Limited mechanisms for providing feedback or involving women in health care decision-making discouraged women from returning for services. Additionally, weak accountability within primary health care systems exacerbated these issues, with poor enforcement of gender-equitable policies, inadequate community feedback mechanisms, and minimal representation of women in the health care leadership roles (Fig 2). A detailed analysis of these barriers is presented elsewhere [22,23].

### Gender integration strategies

Informed by the findings from empathic research and co-design consultations with stakeholders, the project developed a comprehensive gender integration strategy to address systemic barriers and promote equitable health and social outcomes (Fig 2). This strategy centered on three key objectives: improving equitable access, enhancing service quality, and strengthening accountability in RMNCH services.

To ensure equitable access, the strategy tailored RMNCH services to meet the unique needs of women. Key interventions included improving access to information and services, participatory women’s group learning and action, and fostering stronger community engagement to build trust and increase service utilization.

To improve the quality of essential health services, the strategy prioritized operationalizing PHC gender-responsive service standards, developing gender-intentional tools and guidelines, and aligning national and sectoral policies through collaboration and networking. These efforts aimed to ensure service delivery is both inclusive and responsive to the needs of women.

Finally, the strategy highlighted the importance of technical oversight and accountability by conducting regular follow-up, fostering cross-sectoral collaboration and partnerships, and implementing gender-responsive monitoring and evaluation systems. Together, these strategic objectives established a comprehensive framework for integrating gender into PHC, which would lead to sustainable improvements in health equity and outcomes. These include ensuring no harm, promoting client-responsive care, enhancing positive client experiences, strengthening women's agency, and advancing gender integration. Specific details of the gender-integration strategies and activities are described below.

**Capacity building:** To enhance gender integration in project implementation, a comprehensive capacity-building strategy was undertaken. On May 23–24, 2022, 15 project staff participated in an interactive gender training organized by the Global Center for Gender Equality. This training enabled the team to identify key gender integration opportunities and strategies, including conducting gender analysis, building staff capacity, ensuring gender-balanced team composition, and embedding gender considerations into core project strategies such as community engagement (n = 11,059), PLA (n = 265) and lifesaving package delivery group (n = 187), quality improvement, community scorecards, and monitoring and evaluation systems.

Following the training, gender was systematically integrated into the project’s monitoring and evaluation (M&E) system and implementation plan. A cascade training approach was used to expand the reach of gender capacity building. A total of 35 training of trainers were trained at the national and regional levels, who then facilitated the training of 305 staff (36% female) across the project intervention woredas. These efforts strengthened the capacity of staff and stakeholders to incorporate gender-sensitive approaches in community engagement, education, and service delivery, laying the foundation for more inclusive and equitable project outcomes.

**Community engagement:** Community engagement was central, empowering local populations to actively participate in decision-making, designing intervention, implementing and evaluation outcome and impact of health services. Over 11,059 VHLs, more than half of whom were women (51%), were recruited to strengthen community participation, promote men's involvement in maternal health decisions, and empower women through participatory approaches.

**Fig 3:**
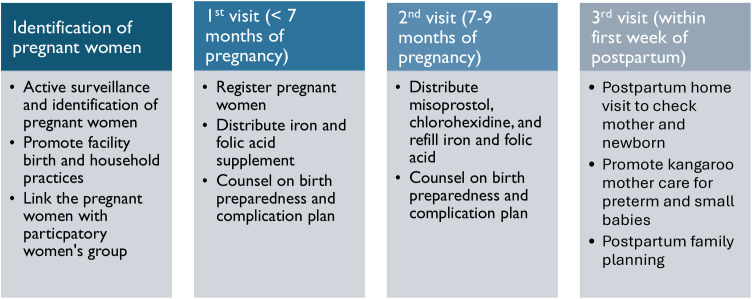
Community-based delivery of MNH services and home visitation schedule for vulnerable communities.

**Community-based distribution and delivery of MNH interventions for vulnerable women:** The community-based delivery of MNH services was expanded to reach vulnerable populations with high home birth rates, emphasizing gender inclusivity (Fig 3). Since December 2023 to June 2025, JSI and Amref jointly implemented home-based interventions such as promoting facility births, educating communities on birth preparedness and complication readiness, and facilitating the advance distribution of iron and folic acid (IFA) supplementation, misoprostol, chlorhexidine, and progesterone-only family planning pills. These efforts are being carried out at seven health centers and across 38 Kebeles (sub-district) with vulnerable communities through home visits conducted by community volunteers to address barriers related to geographic distance and lack of facility access.

**Participatory women’s groups for MNH:** Participatory Learning and Action (PLA) methods have been employed to promote community-driven solutions, complemented by the development of gender-sensitive community scorecards to monitor and assess services from a gender perspective. Since March 2023, PLA has been implemented in four woredas across 41 Kebeles with Vulnerable communities (6 agrarian and 35 pastoral) in the Afar, Somali, and Sidama regions, and reached 96, 476. Of these, 69.5% (67,013) of them were women and girls who attended the 11 cycles of PLA approaches.

**Gender-responsive health service delivery:** Since March 2023, a combined community-facility quality improvement process has been implemented, incorporating gender integration into the planning, monitoring, and evaluation stages. This included the development of gender-sensitive indicators, such as measures of respectful maternity care, women’s involvement in clinical decision-making, and male partner involvement in antenatal care, and gender-intentional tools, including community scorecards, client exit interview guides, and supportive supervision checklists, to systematically track progress and inform continuous quality improvement. Furthermore, in pastoral settings, a mobile health service delivery model was designed and implemented to address the health needs and demands of underserved communities, including women, children, adolescents, elders, and persons with disabilities.

**Gendered community scorecards:** To integrate gender considerations into the community scorecard process, we ensured equitable engagement of girls, women, boys, and men as both members and leaders in client councils. These councils play a crucial role in providing community feedback on PHC services, promoting inclusivity and diverse perspectives in service evaluation and improvement.

**Monitoring and evaluation:** A robust monitoring and evaluation system was established to track the success and impact of these gender integration efforts, enabling continuous adaptation and improvement. To support these initiatives, the project developed practical resources, including a gender integration guide, training manuals, and self-assessment tools, which are available upon request, aimed at building both institutional and individual capacity. By equipping staff with the tools and knowledge to incorporate gender considerations into their work and advocating for policies that promote equity, the project has fostered lasting impact and resilience in addressing gender disparities within health systems.

### Gender integration frameworks

**Fig 4:**
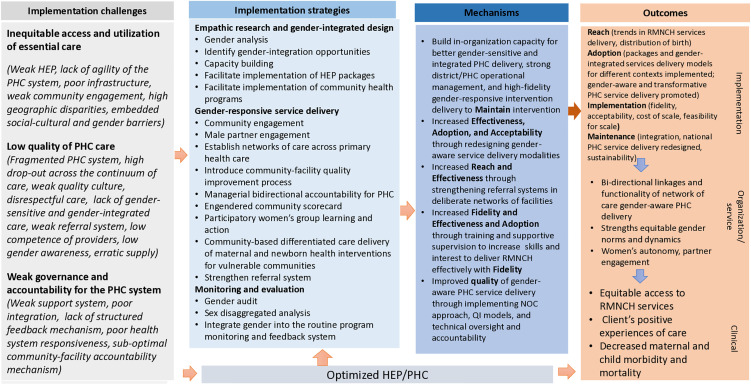
Gender-integration framework across primary health care service delivery.

During the project design phase, we developed a program theory of change hypothesizing that strengthen the health system capacity to pressure-test HEP/PHC service delivery approaches and modalities to strengthen the functionality of and bidirectional linkage across PHC delivery platforms for improved RMNCH outcomes. Building on the findings from the gender analysis and co-design process for integrating gender across PHC, we subsequently developed a framework for a gender-responsive, equitable, and high-quality PHC service delivery model that addresses the unique needs of women (Fig 4). The framework for a gender-responsive, equitable, and high-quality PHC service delivery model was developed to address systemic challenges and promote improved health and social outcomes. It focused on strengthening the health system’s capacity to deliver integrated, gender-sensitive services by enhancing organizational management, building provider skills, and implementing tailored service delivery approaches. The model emphasized bi-directional linkages and functional networks of care to ensure women’s unique needs are met through gender-aware service delivery, partner engagement, and effective coverage of RMNCH services.

Key components of the framework included integrating gender considerations at all levels of service delivery. Strategies such as participatory women’s group learning, gender-responsive community scorecards, and strengthened referral systems were designed to enhance community engagement and accountability. Additionally, capacity-building initiatives—including gender-focused training, supportive supervision, and monitoring systems with sex-disaggregated data—were implemented to ensure the sustainability of gender-integrated PHC. Together, these strategies aim to address inequitable access, low-quality care, and weak governance, transforming PHC into a more inclusive, effective, and equitable system.

### Empowered women and communities

Lessons from integrating gender considerations into program design and implementation highlight the transformative impact of inclusive, context-specific strategies in enhancing women’s agency and increasing the uptake of RMNCH services. Gender-sensitive practices, such as home-based care provided by community volunteers, alongside gender-transformative strategies like PLA groups, played a pivotal role in empowering women.

Between March and October 2024, 265 PLA-MNH facilitators were trained (123 in agrarian areas and 142 in pastoral areas). Consequently, a total of 242 PLA-MNH groups were formed (123 in agrarian and 119 in pastoral) with 62% of facilitators being female who are being invested in engaging all community members in RMNCH interventions. Male involvement was further strengthened through targeted PLA groups and community engagement activities. By involving men as allies and decision-makers, the program fostered improved household-level collaboration on health issues and supported women’s health-seeking behaviors.

These interventions empowered women to take an active role in health-related decisions, boosting their autonomy and confidence in managing RMNCH needs. Community involvement and male engagement were critical in promoting shared responsibility for health outcomes.

The household surveys DiD analysis indicated a statistically significant improvement in women’s agencies in the PLA intervention areas. In agrarian areas, the intervention group showed a marked increase from 29.6% at baseline to 45.7% at end-line (a 16.1 percentage point increase, p < 0.001), while the comparison group exhibited only a modest and non-significant rise of 2.5 percentage points (p = 0.154). This yielded a significant DiD effect of +13.6 percentage points (95% CI: 9.65–17.95; p < 0.001). In contrast, although pastoral areas also experienced increases in women’s agency in both the intervention (17.2 percentage points, p < 0.01) and comparison groups (14.6 percentage points, p < 0.001), the net intervention effect was small and not statistically significant (DiD: + 2.6 percentage points; 95% CI: -1.45–6.71; p = 0.206.

### Improved uptake of MNH services

Between December 2023 and June 2025, a total of 3,980 pregnant women—2,439 from agrarian and 1,541 from pastoral communities—were followed across 38 kebeles, representing roughly half of expected pregnancies (75% in agrarian and 33% in pastoral areas). Among the 1,867 women who gave birth during the intervention, institutional delivery rates were 38% in agrarian and 31% in pastoral areas. Among home births, 92% of women used misoprostol for postpartum hemorrhage prevention (89% agrarian, 97% pastoral), and chlorhexidine cord care was applied by 82% overall (77% agrarian, 89% pastoral).

The household survey DiD analysis indicated that the community-based MNH delivery strategy led to improvements in institutional delivery in both agrarian and pastoral settings compared with their respective comparison groups. Because institutional delivery rates differed between intervention and comparison areas at baseline, particularly in the agrarian setting where comparison areas had substantially higher coverage, the DiD estimates are interpreted as the differential change over time rather than differences in absolute coverage levels. In agrarian areas, institutional delivery increased significantly from 17.2% at baseline to 25.1% at end-line (p = 0.026), while the comparison group showed a non-significant decline from 75.7% to 67.8% (p = 0.140), resulting in a substantial and statistically significant DiD of +15.8 percentage points (p < 0.001). Similarly, in pastoral settings, institutional delivery more than doubled in the intervention group from 12.1% to 25.8% (p = 0.002), whereas the comparison group showed a modest and non-significant increase from 18.9% to 20.3% (p = 0.598), yielding a significant DiD of +12.3 percentage points (p = 0.031).

For the PLA strategy, institutional delivery increased substantially in the intervention group from 72.3% to 92.4% (p < 0.001). In contrast, pastoral areas exhibited strong positive effects, with institutional delivery rising sharply in the intervention group from 12.6% to 42.5% (p < 0.001), compared to an increase from 19.9% to 33.9% (p < 0.001) in the comparison group, translated into a significant positive DiD of +14.0 percentage points (p < 0.001) (Table 2).

**Table 2:** Changes in the uptake of institutional delivery by program domain and agrarian and pastoral settings, August-October 2023 and June-October 2025.

Program	Setting	Intervention			Comparison			DiD	
Baseline	End-line	p-value	Baseline	End-line	p-value	DiD	p-value
Community-based delivery of MNH services	Agrarian	17.2	25.1	0.026	75.7	67.8	0.140	**+15.8**	**<0.001**
	Pastoral	12.1	25.8	0.002	18.9	20.3	0.598	**+12.3**	**0.031**
PLA	Agrarian	72.3	92.4	<0.001	73.9	65.0	0.010	**+29.0**	**0.010**
	Pastoral	12.6	42.5	<0.001	19.9	33.9	<0.001	**+15.9**	**<0.001**

## Discussion

The project followed a thorough, participatory, and evidence-based design process, grounded in formative empathic inquiry with human-centered design and gender analysis, and iterative co-creation with key stakeholder consultations. The human-centered design approach intentionally placed end-users at the center of the design process to ensure that interventions were contextually relevant, acceptable, feasible, and responsive within routine primary health care settings. This was operationalized through formative empathic inquiry using qualitative interviews, focus group discussions, community mapping, and service delivery observations, with findings synthesized into user journeys, personas, and problem statements that informed solution design, followed by co-creation workshops with stakeholders to support rapid prototyping and iterative refinement of implementation strategies through continuous feedback and contextual validation. This approach led to the development of comprehensive, gender-integrated strategies and frameworks aimed at addressing persistent challenges in the delivery and utilization of high-impact RMNCH practices. Subsequently, we developed a framework for a gender-responsive, equitable, and high-quality PHC service delivery model that addresses the unique needs of women. The integration of gender considerations into PHC service delivery has notably enhanced women’s agency, improving their access to maternal and newborn health services.

The findings of this project align with prior research, which has documented the disproportionate impact of gender norms, limited autonomy, societal expectations, and structural barriers on women’s health outcomes [10,24,25]. This project builds on existing knowledge by offering a comprehensive strategy to address these challenges, emphasizing capacity building, community engagement, and service quality improvement. Previous studies found that community-based interventions and the active involvement of health workers in delivering gender-sensitive care are essential in overcoming demand-side barriers such as geographic distance, financial constraints, and restrictive cultural norms [10,14,24,25].

The project’s learnings highlight the critical role of gender-transformative approaches, particularly participatory women’s group learning and action cycles, in empowering women to make informed health decisions and address their own health needs. Evidence shows that women’s group discussions notably enhance women’s agency and improve maternal and newborn health outcomes, especially in low-resource settings [26,27]. Additionally, gender-intentional, community-based delivery of MNH services by community volunteers has proven effective in improving access to and utilization of essential health services for vulnerable women. These programs empower women by providing them with the knowledge, skills, and decision-making opportunities related to their own and their families’ health, thereby boosting their agency to access MNH services and adopt healthier practices [28,29]. A review of evidence on gender integration in RMNCH programs showed the effectiveness of both gender-accommodating and gender-transformative approaches [9]. To facilitate scale-up and sustainability, these community-based approaches should be institutionalized within existing primary health care and community health systems through integration into routine community health worker platforms, strengthened supervision and mentorship mechanisms, incorporation into national RMNCH guidelines, and continued community ownership and engagement.

Gender-transformative strategies not only on empowering women but also on engaging men as allies in RMNCH create a supportive environment for better health outcomes. These results align with previous literature suggesting that involving men in maternal health decision-making can reduce women's burden, promote shared responsibility for RMNCH outcomes, and address gender influences on maternal and child health [9,10,14,24,25,30]. However, male engagement is often constrained by sociocultural norms that view pregnancy and childbirth as women's responsibilities, limited awareness of men's roles in maternal health, competing work commitments, and health services that may not be welcoming to male participation. In this study, these barriers were addressed through gender-transformative community engagement approaches, including participatory learning and action groups, community dialogues, and the engagement of community volunteers to promote men's involvement in birth preparedness, maternal health decision-making, and care-seeking. Male engagement in maternal health through couple education, community dialogues, and male support groups enhances women’s agency and improves maternal and newborn health outcomes by fostering shared decision-making, increasing access to care, and providing emotional and financial support [30,31].

Gender-responsive PHC service delivery, including quality improvement strategies, gender training, M&E, and community scorecards, strengthens women's agency and improves maternal and newborn health outcomes. Quality improvement initiatives, such as standards-based management, boost the uptake of essential maternal care services, while gender-sensitive M&E practices address disparities in service delivery, ensuring equitable access for all [32,33]. Community scorecards and gender training for health care workers empower women, improve health-seeking behaviors, and promote respectful, personalized care, ultimately reducing maternal and child mortality [12,34]. International health agencies also emphasize integrating gender equality into PHC as a key strategy for achieving universal health coverage. By addressing gender dynamics and promoting gender equality, PHC systems can become more responsive to the needs of women, making them more inclusive, effective, and sustainable [12].

This study highlights the transformative potential of gender-focused strategies in overcoming health inequities and the positive influence on accessing and delivering RMNCH services at the PHC level. It also provides insights for designing gender-intention strategies and frameworks aimed at enhancing PHC service delivery. As a comprehensive synthesis, it emphasizes the integration of gender in PHC, aiming to empower women to utilize RMNCH services effectively. These interventions provide valuable lessons for other regions aiming to implement gender-responsive, community-based health programs. Despite its strengths, the study has limitations. The effectiveness of the proposed gender-integration strategies and framework are not evaluated through a rigorous research design, which limits the ability to assess their impact and effectiveness in practice. Future studies should employ robust implementation and effectiveness research designs, including hybrid implementation-effectiveness and quasi-experimental approaches, to assess intervention effectiveness, scalability, sustainability, cost-effectiveness, and their influence on women's agency, gender norms, health service utilization, and RMNCH outcomes across diverse settings.

## Conclusions

This study highlights the importance of integrating gender considerations into the design, implementation, and monitoring of health services to address the unique challenges faced by women, men, girls, and boys in the PHC system. Drawing on lessons learned, it is vital to ensure that these efforts contribute to the sustainability of gender-responsive practices while promoting greater male involvement in maternal health decision-making. These initiatives are crucial for assessing the impacts of gender integration in PHC on health and social outcomes for all genders. Furthermore, they enable the health system to track progress toward achieving universal health coverage and gender equity, ultimately driving measurable improvements in health outcomes for women, girls, and gender-diverse individuals.

Abbreviations: DiD, Difference-in-difference; IPHCSD, Improve Primary Health Care Service Delivery; IDI, in-depth interview; IFA, iron and folic acid; FGD, focus group discussion; GoE, Government of Ethiopia; HEP, Health Extension Program; KII, key informant interview; M&E, monitoring and evaluation; MNH, maternal and newborn health; MOH, Ministry of Health; NoCs, Networks of Care; PHC, primary health care; PLA, Participatory Learning and Action; RMNCH, reproductive, maternal, newborn, and child health; VHL,Village Health Leader.

## Supporting Information

S1 File Survey dataset. This file contains the survey data with variables and their values used for analysis.
(CSV)

S2 File Survey questionnaire. This file contains the survey questionnaires used to collect information from study participants. The first sheet provides variable definitions (data dictionary) in English and local languages (Amharic, Afaan Oromo, and Af-Somali), while the second sheet contains the answer choices for each variable.
(XLSX)

## References

[1] WHO. Trends in maternal mortality 2000 to 2023: estimates by WHO, UNICEF, UNFPA, World Bank Group and UNDESA/Population Division. Geneva: World Health Organization. 2025.

[2] Duke T. Levels and trends in child mortality estimation. BMJ Publishing Group Ltd. 2024. 10.1136/archdischild-2024-327211 38631885

[3] Aga MA, Chen D-G. Maternal mortality in Ethiopia (2015-2025): a systematic review of recent evidence and determinants. BMC Public Health. 2025;26(1):539. doi: 10.1186/s12889-025-26101-w 41462176 PMC12888282

[4] Gudayu TW. Epidemiology of neonatal mortality: a spatial and multilevel analysis of the 2019 mini-Ethiopian demographic and health survey data. BMC Pediatr. 2023;23(1):26. doi: 10.1186/s12887-023-03838-0 36647037 PMC9843859

[5] Manandhar M, Hawkes S, Buse K, Nosrati E, Magar V. Gender, health and the 2030 agenda for sustainable development. Bull World Health Organ. 2018;96(9):644–53. doi: 10.2471/BLT.18.211607 30262946 PMC6154065

[6] Mosha I, Ruben R, Kakoko D. Family planning decisions, perceptions and gender dynamics among couples in Mwanza, Tanzania: a qualitative study. BMC Public Health. 2013;13:523. doi: 10.1186/1471-2458-13-523 23721196 PMC3679800

[7] Downe S, Finlayson K, Oladapo OT, Bonet M, Gülmezoglu AM. What matters to women during childbirth: A systematic qualitative review. PLoS One. 2018;13(4):e0194906. doi: 10.1371/journal.pone.0194906 29664907 PMC5903648

[8] Finlayson K, Downe S. Why do women not use antenatal services in low- and middle-income countries? A meta-synthesis of qualitative studies. PLoS Med. 2013;10(1):e1001373. doi: 10.1371/journal.pmed.1001373 23349622 PMC3551970

[9] Kraft JM, Wilkins KG, Morales GJ, Widyono M, Middlestadt SE. An evidence review of gender-integrated interventions in reproductive and maternal-child health. J Health Commun. 2014;19 Suppl 1(sup1):122–41. doi: 10.1080/10810730.2014.918216 25207450 PMC4205884

[10] Morgan R, Tetui M, Muhumuza Kananura R, Ekirapa-Kiracho E, George AS. Gender dynamics affecting maternal health and health care access and use in Uganda. Health Policy Plan. 2017;32(suppl_5):v13–21. doi: 10.1093/heapol/czx011 29244103 PMC5886085

[11] Kebede Y, Teshome F, Binu W, Kebede A, Seid A, Kasaye HK, et al. Structural, programmatic, and sociocultural intersectionality of gender influencing access-uptake of reproductive, maternal, and child health services in developing regions of Ethiopia: A qualitative study. PLoS One. 2023;18(3):e0282711. doi: 10.1371/journal.pone.0282711 36881602 PMC10045587

[12] Faramand T, Ivankovich M, J H. A Guide to Integrating Gender in Improvement. Chevy Chase MD: University Research Co., LLC. 2017.

[13] USAID. A manual for integrating gender into reproductive health and HIV programs. USAID. 2009.

[14] Eregata GT, Hailu A, Geletu ZA, Memirie ST, Johansson KA, Stenberg K, et al. Revision of the Ethiopian Essential Health Service Package: An Explication of the Process and Methods Used. Health Syst Reform. 2020;6(1):e1829313. doi: 10.1080/23288604.2020.1829313 33300838

[15] MOH. National gender mainstreaming manual for health. Addis Ababa, Ethiopia: Ministry of Health. 2021.

[16] Tiruneh GT, Fesseha N, Ayehu T, Chitashvili T, Argaw MD, Shiferaw BB, et al. Networks of care for optimizing Primary Health Care Service Delivery in Ethiopia: Enhancing relational linkages and care coordination. PLoS One. 2025;20(1):e0314807. doi: 10.1371/journal.pone.0314807 39752410 PMC11698449

[17] MOH. Realizing universal health coverage through primary health care: a roadmap for optimizing the Ethiopian health extension program 2020 - 2035. Addis Ababa, Ethiopia: Ministry of Health, Ethiopia. 2020.

[18] Kalaris K, Wong G, English M. Understanding networks in low-and middle-income countries’ health systems: A scoping review. PLOS Glob Public Health. 2023;3(1):e0001387. doi: 10.1371/journal.pgph.0001387 36962859 PMC10022031

[19] WHO. Networks of care for maternal and newborn health: implementation guidance. World Health Organization. 2024.

[20] WHO. Gender mainstreaming for health managers: a practical approach. Geneva: World Health Organization. 2011.

[21] EngenderHealth. Global strategy for gender-transformative programs. New York: EngenderHealth. 2022.

[22] Semahegn A, Mehretie Adinew Y, Tiruneh GT, Argaw MD, Bekele B, Tesfaye M, et al. Gender disparities and barriers to access and use of essential health services in Ethiopia: Designing primary health care through gender lens. PLOS Glob Public Health. 2025;5(6):e0004813. doi: 10.1371/journal.pgph.0004813 40540512 PMC12180718

[23] Semahegn A, Tiruneh GT, Mirkuzie AH, Argaw MD, Fesseha N, Teferi M, et al. Gender dynamics affecting women’s uptake of maternal health service in agrarian and pastoral settings in Ethiopia: a community-based cross-sectional study. Reprod Health. 2026;23(1):98. doi: 10.1186/s12978-026-02325-w 41928251 PMC13169562

[24] Jones N, Pincock K, Baird S, Yadete W, Hamory Hicks J. Intersecting inequalities, gender and adolescent health in Ethiopia. Int J Equity Health. 2020;19(1):97. doi: 10.1186/s12939-020-01214-3 32539778 PMC7296636

[25] Ambrose N, Leonard B, Kor JA-NM, Sumah AN, Zwanikken P. The underlying gendered factors influencing access to and utilization of skilled birth attendance (Sba): A case study in Ghana. Advances in Social Sciences Research Journal. 2022;9(7).

[26] Prost A, Colbourn T, Seward N, Azad K, Coomarasamy A, Copas A, et al. Women’s groups practising participatory learning and action to improve maternal and newborn health in low-resource settings: a systematic review and meta-analysis. Lancet. 2013;381(9879):1736–46. doi: 10.1016/S0140-6736(13)60685-6 23683640 PMC3797417

[27] Seward N, Neuman M, Colbourn T, Osrin D, Lewycka S, Azad K, et al. Effects of women’s groups practising participatory learning and action on preventive and care-seeking behaviours to reduce neonatal mortality: A meta-analysis of cluster-randomised trials. PLoS Med. 2017;14(12):e1002467. doi: 10.1371/journal.pmed.1002467 29206833 PMC5716527

[28] Olaniran A, Madaj B, Bar-Zev S, van den Broek N. The roles of community health workers who provide maternal and newborn health services: case studies from Africa and Asia. BMJ Glob Health. 2019;4(4):e001388. doi: 10.1136/bmjgh-2019-001388 31478012 PMC6703286

[29] Maluka SO, Mpambije CJ, Kamuzora PC, Fitzgerald S. The effects of community-based interventions on the uptake of selected maternal and child health services: experiences of the IMCHA project in Iringa Tanzania, 2015-2020. BMC Pregnancy Childbirth. 2023;23(1):328. doi: 10.1186/s12884-023-05638-x 37158851 PMC10165785

[30] Comrie-Thomson L, Tokhi M, Ampt F, Portela A, Chersich M, Khanna R. Challenging gender inequity through male involvement in maternal and newborn health: critical assessment of an emerging evidence base. Culture, health & sexuality. 2015;17(sup2):177–89. 10.1080/13691058.2015.1053412 PMC4706017 26159766

[31] Yargawa J, Leonardi-Bee J. Male involvement and maternal health outcomes: systematic review and meta-analysis. J Epidemiol Community Health. 2015;69(6):604–12. doi: 10.1136/jech-2014-204784 25700533 PMC4453485

[32] Sophie G, Valerie B-A, Cornelia B. Quality improvement in maternal and newborn healthcare: lessons from programmes supported by the German development organisation in Africa and Asia. BMJ Global Health. 2019;4(5):e001562. doi: 10.1136/bmjgh-2019-001562 PMC6747907 31565404

[33] Hagaman AK, Singh K, Abate M, Alemu H, Kefale AB, Bitewulign B, et al. The impacts of quality improvement on maternal and newborn health: preliminary findings from a health system integrated intervention in four Ethiopian regions. BMC Health Serv Res. 2020;20(1):522. doi: 10.1186/s12913-020-05391-3 32513236 PMC7282234

[34] Lindsay S, Rezai M, Kolne K, Osten V. Outcomes of gender-sensitivity educational interventions for healthcare providers: A systematic review. Health Education J. 2019;78(8):958–76. doi: 10.1177/0017896919859908

