# Supplementary material for: Advancing gender equity in primary health care: Lessons from integrating gender-responsive strategies into service delivery in Ethiopia
Source: PLOS Glob Public Health. 2026 Jul 16;6(7):e0006764. doi: 10.1371/journal.pgph.0006764 (PMC13375001; doi:10.1371/journal.pgph.0006764)
Supplement: Attachment — Submitted filename: PGPH-D-26-01529.review.ch.docx [file pgph.0006764.s003.docx]

PLOS Reviewer’s Comments: PGPH-D-26-01529

05/27/2026

This study, *Advancing Gender Equity in Primary Health Care: Lessons from Integrating Gender-Responsive Strategies into Service Delivery in Ethiopia,* describes an impressive initiative to improve gender equity in maternal and child health services and outcomes.

This study was conducted by a robust team of authors with deep roots in the field. They are familiar with Ethiopian health system priorities and designed their study and interventions to accelerate gender equity.

The robust study design included “systematic, evidence-based quasi-experimental embedded implementation research with participatory multi-method approaches to integrate gender considerations into PHC. The process included before-and-after controlled household studies, phenomenological research, gender analysis, and stakeholder engagement to identify barriers and opportunities in addressing gender disparities in health care access.”

Findings from participant surveys, qualitative interviews and focus groups guided gender-intentional strategies to improve health service delivery. Research and interventions were tied to the Improve Primary Health Care Service Delivery (IPHCSD) project to strengthen the country’s PHC system and enhance RMNCH outcomes. These interventions, linked with evaluation, monitoring and accountability, have the potential to transform the Ethiopian PHC system and to improve RMNCH.

Congratulations on this outstanding study which effectively describes the “transformative potential of gender-focused strategies in overcoming health inequities and the positive influence on accessing and delivering RMNCH services at the PHC level.”

**Following are suggestions for minor edits and/or improvements; numbers indicate lines in the text:**

64: To set the stage, provide maternal and infant mortality statistics for Ethiopia including regional variations.

79: “the systematic integration of gender considerations into RMNCH programs remains limited” in the discussion, revisit this statement with suggestions for how these findings might be applied and extended nation-wide.

86: Change *identifies to identified* to make the verbs consistent.

94: Reverse the order of primary care system components from “primary level includes primary hospitals, health centers, and health posts” to the opposite. Most conventions describe PHC as starting from the lowest level of health posts; in some contexts, hospitals are considered as secondary levels of care.

139: It would be helpful to provide a summary of empathic formative research findings in an appendix.

147: Elaborate on the human-centered design approaches, either in the introduction or later in the discussion.

175: Describe or display key elements of the action plan.

196: Change strengthened to strengthen.

211-232: Gender-related barriers and strategies are critical to the study. Well done!

269: Bravo for recognizing the crucial importance of community participation and for outreach to vulnerable populations.

292: Provide examples of gender-sensitive indicators and tools; could be in appendix or see next comment.

305: Provide references or access to resources on request.

332: Figure 4 is powerful and could be used in trainings throughout the health system.

368: Why is there such a big difference between institutional delivery rates between the intervention and comparison groups? The reported rates are confusing.

403:  Address how gender-intentional, community-based delivery of MNH services by community volunteers can be expanded and sustained.

414: Discuss barriers to male engagement and how this study addressed them.

434: Suggest strategies to expand and further study the outcomes of these interventions.
