# Supplementary material for: Advancing gender equity in primary health care: Lessons from integrating gender-responsive strategies into service delivery in Ethiopia
Source: PLOS Glob Public Health. 2026 Jul 16;6(7):e0006764. doi: 10.1371/journal.pgph.0006764 (PMC13375001; doi:10.1371/journal.pgph.0006764)
Supplement: Attachment — Submitted filename: Point-by-point response to editor and reviewers.docx [file pgph.0006764.s005.docx]

**Point-by-point response to editorial and reviewers**

PGPH-D-26-01529: Advancing Gender Equity in Primary Health Care: Lessons from Integrating Gender-Responsive Strategies into Service Delivery in Ethiopia

Dear Editor,

We would like to express our sincere gratitude to the reviewers for their insightful and constructive comments, which have significantly strengthened the manuscript. Below, we provide point-by-point responses to each comment, with corresponding revisions highlighted in red in the revised manuscript. We have also carefully reviewed the manuscript to ensure it aligns with the journal’s formatting and stylistic requirements.

Journal Requirements:

1. Please ensure that your Ethics Statement is available in its entirety at the beginning of your Methods section, under a subheading 'Ethics Statement'.

**Response**: Thank you for this comment. We have revised the manuscript and moved the Ethics Statement to the Methods section under a dedicated subheading titled “Ethics Statement,” in accordance with journal requirements.

2. The resolution of Figures 1-4 are very low and somewhat difficult to read. It is important that our Editors and Peer Reviewers are able to read all parts of a submission. Please replace these figures with higher resolution copies.

**Response**: Thank you for this observation. We have replaced Figures 1–4 with higher-resolution versions.

3. We note that your Data Availability Statement is currently as follows: "The dataset used and analyzed during this study is included as supplementary information to this article (S1 File), along with the interview guide (S2 File)."

 Please confirm at this time whether or not your submission contains all raw data required to replicate the results of your study. Authors must share the “minimal data set” for their submission. PLOS defines the minimal data set to consist of the data required to replicate all study findings reported in the article, as well as related metadata and methods (<https://journals.plos.org/plosone/s/data-availability#loc-minimal-data-set-definition>).
 
 For example, authors should submit the following data:
 
 - The values behind the means, standard deviations and other measures reported;
 - The values used to build graphs;
 - The points extracted from images for analysis.
 
 Authors do not need to submit their entire data set if only a portion of the data was used in the reported study.
 
**Response**: Thank you for this important clarification. We confirm that we have revised the Data Availability Statement and provided the minimal dataset required to replicate the study findings.

If the reviewer comments include a recommendation to cite specific previously published works, please review and evaluate these publications to determine whether they are relevant and should be cited. There is no requirement to cite these works unless the editor has indicated otherwise. 

Please review your reference list to ensure that it is complete and correct. If you have cited papers that have been retracted, please include the rationale for doing so in the manuscript text, or remove these references and replace them with relevant current references. Any changes to the reference list should be mentioned in the rebuttal letter that accompanies your revised manuscript. If you need to cite a retracted article, indicate the article’s retracted status in the References list and also include a citation and full reference for the retraction notice.

**Response**: We have carefully reviewed and updated the reference list accordingly. All citations have been checked for accuracy and completeness, and any unpublished or non-peer-reviewed sources have been removed to ensure compliance with journal standards.

**Reviewer #1:** 
This study, Advancing Gender Equity in Primary Health Care: Lessons from Integrating Gender-Responsive Strategies into Service Delivery in Ethiopia, describes an impressive initiative to improve gender equity in maternal and child health services and outcomes.

This study was conducted by a robust team of authors with deep roots in the field. They are familiar with Ethiopian health system priorities and designed their study and interventions to accelerate gender equity.

The robust study design included “systematic, evidence-based quasi-experimental embedded implementation research with participatory multi-method approaches to integrate gender considerations into PHC. The process included before-and-after controlled household studies, phenomenological research, gender analysis, and stakeholder engagement to identify barriers and opportunities in addressing gender disparities in health care access.”

Findings from participant surveys, qualitative interviews and focus groups guided gender-intentional strategies to improve health service delivery. Research and interventions were tied to the Improve Primary Health Care Service Delivery (IPHCSD) project to strengthen the country’s PHC system and enhance RMNCH outcomes. These interventions, linked with evaluation, monitoring and accountability, have the potential to transform the Ethiopian PHC system and to improve RMNCH.

Congratulations on this outstanding study which effectively describes the “transformative potential of gender-focused strategies in overcoming health inequities and the positive influence on accessing and delivering RMNCH services at the PHC level.”

Following are suggestions for minor edits and/or improvements; numbers indicate lines in the text:

**Response**: We sincerely thank Reviewer #1 for the thorough and constructive review and for the positive assessment of our work. We appreciate the insightful suggestions, which have helped improve the clarity and quality of the manuscript.
**- Introduction:**

- Line 64: To set the stage, provide maternal and infant mortality statistics for Ethiopia including regional variations.

**Response**: Maternal and newborn mortality statistics, including regional variations, have been added to strengthen the background and contextualize the study. **Lines 62-66**

- Line 79: “the systematic integration of gender considerations into RMNCH programs remains limited” in the discussion, revisit this statement with suggestions for how these findings might be applied and extended nation-wide.

**Response**: We have expanded the discussion to include how the study findings can inform national-scale application of gender integration within Ethiopia’s primary health care system, including policy alignment, integration into routine PHC platforms, and scale-up considerations. **Lines 458-462**

- Line 86: Change identifies to identified to make the verbs consistent.

**Response**: This correction has been made.

**- Methods:**

- Line 94: Reverse the order of primary care system components from “primary level includes primary hospitals, health centers, and health posts” to the opposite. Most conventions describe PHC as starting from the lowest level
of health posts; in some contexts, hospitals are considered as secondary levels of care.

**Response**: This has been revised to reflect the correct PHC structure, starting from health posts, followed by health centers and primary hospitals. **Lines 115**

- Line 139: It would be helpful to provide a summary of empathic formative research findings in an appendix.

**Response**: Thank you for this suggestion. A summary of key formative/empathic research findings has been included in Table 1, and relevant supporting publications are also cited in the manuscript. **Line 180**

- Line 147: Elaborate on the human-centered design approaches, either in the introduction or later in the discussion.

**Response**: We have elaborated the human-centered design approach in the revised manuscript within the Discussion section, providing additional detail on the participatory, iterative co-creation process used to inform intervention design. **Line 428-440**

- Line 175: Describe or display key elements of the action plan.

Response: This has been addressed by clearly outlining the key components of the action plan, including strategies for improving equitable access, enhancing service quality, and strengthening accountability within gender-responsive PHC implementation. **Line 190-194**

- Line 196: Change strengthened to strengthen.

Response: This correction has been made.

**- Results:**

- Lines 211-232: Gender-related barriers and strategies are critical to the study. Well done.

**Response**: We sincerely thank the reviewer for this positive feedback.

- Line 269: Bravo for recognizing the crucial importance of community participation and for outreach to vulnerable populations.

**Response**: We sincerely thank the reviewer for this positive feedback.

- Line 292: Provide examples of gender-sensitive indicators and tools; could be in appendix or see next comment.

**Response**: We thank the reviewer for this important suggestion. We have clarified this section by providing examples of the gender-sensitive indicators and tools used in the community–facility quality improvement process. **Lines 331-335**

- Line 305: Provide references or access to resources on request.

**Response**: The relevant tools, guides, and resources supporting the gender integration and M&E processes are available upon request

- Line 332: Figure 4 is powerful and could be used in trainings throughout the health system.

**Response:** We sincerely thank the reviewer for this positive feedback and helpful suggestion regarding the potential use of the figure for training and capacity-building within the health system.

- Line 368: Why is there such a big difference between institutional delivery rates between the intervention and comparison groups? The reported rates are confusing.

**Response:** We thank the reviewer for noting this important point. The intervention and comparison groups were not identical at baseline, particularly in the agrarian setting, where the comparison areas had substantially higher institutional delivery coverage than the intervention areas prior to implementation of the community-based MNH delivery strategy. This reflects contextual and programmatic differences between the selected districts rather than an effect of the intervention itself. The Difference-in-Differences (DiD) approach was used specifically to account for these baseline differences by estimating changes over time between groups rather than comparing absolute levels at a single time point. To improve clarity, we have revised the Results section to explicitly acknowledge this baseline imbalance and added the following sentence:

*“Because institutional delivery rates differed between intervention and comparison areas at baseline—particularly in the agrarian setting where comparison areas had substantially higher coverage—the DiD estimates are interpreted as the differential change over time rather than differences in absolute coverage levels.”* **Lines 407-410**

**- Discussion and conclusion:**

- Line 403: Address how gender-intentional, community-based delivery of MNH services by community volunteers can be expanded and sustained.

**Response**: We thank the reviewer for this important comment. We have expanded the Discussion section to describe pathways for scaling and sustaining gender-intentional, community-based MNH service delivery. **Lines 458-462**

- Line 414: Discuss barriers to male engagement and how this study addressed them.

**Response**: We thank the reviewer for this valuable suggestion. We have expanded the Discussion section to acknowledge common barriers to male engagement in RMNCH and describe how the project addressed these barriers through gender-transformative community engagement approaches. **Lines 467-473**

- Line 434: Suggest strategies to expand and further study the outcomes of these interventions.

**Response**: We thank the reviewer for this suggestion. We have expanded the Discussion section to include recommendations for scaling and further evaluating these interventions. **Lines 495-499**


**Reviewer #2**

Introduction: The section will benefit by defining gender integration paradigms at the outset. Then, it is also good to highlight the integration of gender into primary healthcare practices.

**Response**: Response: We thank the reviewer for this helpful suggestion. In response, we revised the Introduction to define key gender integration paradigms, including gender-accommodating and gender-transformative approaches along the gender integration continuum, and to clarify their relevance to RMNCH programming. We also strengthened the discussion of how gender can be systematically integrated into primary health care practices—including service delivery, community engagement, quality improvement, and monitoring and evaluation—to improve the responsiveness, equity, and quality of care. These additions provide a clearer conceptual foundation for the gender integration strategies and framework presented in the manuscript. **Lines 74-84 and 131-136**

Objectives: The objective of the study should be formulated by specifically mentioning the lessons learned in the integration of gender into the primary healthcare system of Ethiopia

Response: We thank the reviewer for this suggestion. We have revised the study objective to more explicitly reflect the focus on lessons learned from integrating gender into Ethiopia’s primary health care system. **Lines 98-101**

Methods: What is the theoretical foundation (model) basis for the gender integration in the primary healthcare system of Ethiopia? Is there any use of the well-known gender integration models?

Response: We thank the reviewer for this important question. The gender integration approach in this study is grounded in established gender and implementation science frameworks rather than a single standalone model. Specifically, it draws on the WHO gender mainstreaming approach for health systems, the USAID Gender Integration Continuum, and the EngenderHealth Gender Integration Framework. Given the absence of a single consolidated framework for gender integration in primary health care, the design is further informed by implementation science principles, including human-centered design, participatory co-creation, and systems thinking, which support adaptation to local sociocultural and health system contexts. We have reflected these frameworks and concepts in the revised section “Pathways to gender integration,” where the phased implementation approach is now explicitly aligned with these guiding frameworks. **Lines 131-136**

Results: well written
Discussion: Well written
Conclusion: Well written

**Response**: We sincerely thank the reviewer for the positive feedback.
