# Supplementary material for: Advancing gender equity in primary health care: Lessons from integrating gender-responsive strategies into service delivery in Ethiopia
Source: PLOS Glob Public Health. 2026 Jul 16;6(7):e0006764. doi: 10.1371/journal.pgph.0006764 (PMC13375001; doi:10.1371/journal.pgph.0006764)
Supplement: Peer review history

# Peer review: Decision Letter 0

**DOI:** 10.1371/journal.pgph.0006764.r001

**Authors:** Damen Haile Mariam

11 Jun 2026

PGPH-D-26-01529

Advancing Gender Equity in Primary Health Care: Lessons from Integrating Gender-Responsive Strategies into Service Delivery in Ethiopia

PLOS Global Public Health

Dear Dr. Tiruneh,

Thank you for submitting your manuscript to PLOS Global Public Health. After careful consideration, we feel that it has merit but does not fully meet PLOS Global Public Health’s publication criteria as it currently stands. Therefore, we invite you to submit a revised version of the manuscript that addresses the points raised during the review process.

Please submit your revised manuscript by . If you will need more time than this to complete your revisions, please reply to this message or contact the journal office at globalpubhealth@plos.org. When you're ready to submit your revision, log on to https://www.editorialmanager.com/pgph/ and select the 'Submissions Needing Revision' folder to locate your manuscript file.

Please include the following items when submitting your revised manuscript:

- A letter that responds to each point raised by the editor and reviewer(s). You should upload this letter as a separate file labeled 'Response to Reviewers'.
- A marked-up copy of your manuscript that highlights changes made to the original version. You should upload this as a separate file labeled 'Revised Manuscript with Track Changes'.
- An unmarked version of your revised paper without tracked changes. You should upload this as a separate file labeled 'Manuscript'.

Guidelines for resubmitting your figure files are available below the reviewer comments at the end of this letter.

As the corresponding author, your ORCID iD is verified in the submission system and will appear in the published article. PLOS supports the use of ORCID, and we encourage all coauthors to register for an ORCID iD and use it as well. Please encourage your coauthors to verify their ORCID iD within the submission system before final acceptance, as unverified ORCID iDs will not appear in the published article. *Only*  the individual author can complete the verification step; PLOS staff *cannot*  verify ORCID iDs on behalf of authors.

We look forward to receiving your revised manuscript.

Kind regards,

Damen Haile Mariam, MD, MPH, PhD

Academic Editor

PLOS Global Public Health

Journal Requirements:

1. Please ensure that your Ethics Statement is available in its entirety at the beginning of your Methods section, under a subheading 'Ethics Statement'.

2. The resolution of Figures 1-4 are very low and somewhat difficult to read. It is important that our Editors and Peer Reviewers are able to read all parts of a submission. Please replace these figures with higher resolution copies.

3. We note that your Data Availability Statement is currently as follows: "The dataset used and analyzed during this study is included as supplementary information to this article (S1 File), along with the interview guide (S2 File)."

Please confirm at this time whether or not your submission contains all raw data required to replicate the results of your study. Authors must share the “minimal data set” for their submission. PLOS defines the minimal data set to consist of the data required to replicate all study findings reported in the article, as well as related metadata and methods (https://journals.plos.org/plosone/s/data-availability#loc-minimal-data-set-definition).

For example, authors should submit the following data:

- The values behind the means, standard deviations and other measures reported;

- The values used to build graphs;

- The points extracted from images for analysis.

Authors do not need to submit their entire data set if only a portion of the data was used in the reported study.

If your submission does not contain these data, please either upload them as Supporting Information files or deposit them to a stable, public repository and provide us with the relevant URLs, DOIs, or accession numbers. For a list of recommended repositories, please see https://journals.plos.org/plosone/s/recommended-repositories.

If there are ethical or legal restrictions on sharing a de-identified data set, please explain them in detail (e.g., data contain potentially sensitive information, data are owned by a third-party organization, etc.) and who has imposed them (e.g., an ethics committee). Please also provide contact information for a data access committee, ethics committee, or other institutional body to which data requests may be sent. If data are owned by a third party, please indicate how others may request data access.

If the reviewer comments include a recommendation to cite specific previously published works, please review and evaluate these publications to determine whether they are relevant and should be cited. There is no requirement to cite these works unless the editor has indicated otherwise.

Please review your reference list to ensure that it is complete and correct. If you have cited papers that have been retracted, please include the rationale for doing so in the manuscript text, or remove these references and replace them with relevant current references. Any changes to the reference list should be mentioned in the rebuttal letter that accompanies your revised manuscript. If you need to cite a retracted article, indicate the article’s retracted status in the References list and also include a citation and full reference for the retraction notice.

Additional Editor Comments (if provided):

Reviewer 1 -

- Introduction:

- Line 64: To set the stage, provide maternal and infant mortality statistics for Ethiopia including regional variations.

- Line 79: “the systematic integration of gender considerations into RMNCH programs remains limited” in the discussion, revisit this statement with suggestions for how these findings might be applied and extended nation-wide.

- Line 86: Change identifies to identified to make the verbs consistent.

- Methods:

- Line 94: Reverse the order of primary care system components from “primary level includes primary hospitals, health centers, and health posts” to the opposite. Most conventions describe PHC as starting from the lowest level

of health posts; in some contexts, hospitals are considered as secondary levels of care.

- Line 139: It would be helpful to provide a summary of empathic formative research findings in an appendix.

- Line 147: Elaborate on the human-centered design approaches, either in the introduction or later in the discussion.

- Line 175: Describe or display key elements of the action plan.

- Line 196: Change strengthened to strengthen.

- Results:

- Lines 211-232: Gender-related barriers and strategies are critical to the study. Well done.

- Line 269: Bravo for recognizing the crucial importance of community participation and for outreach to vulnerable populations.

- Line 292: Provide examples of gender-sensitive indicators and tools; could be in appendix or see next comment.

- Line 305: Provide references or access to resources on request.

- Line 332: Figure 4 is powerful and could be used in trainings throughout the health system.

- Line 368: Why is there such a big difference between institutional delivery rates between the intervention and comparison groups? The reported rates are confusing.

- Discussion and conclusion:

- Line 403: Address how gender-intentional, community-based delivery of MNH services by community volunteers can be expanded and sustained.

- Line 414: Discuss barriers to male engagement and how this study addressed them.

- Line 434: Suggest strategies to expand and further study the outcomes of these interventions.

Reviewer 2 -

- Introduction:

- The section will benefit by defining gender integration paradigms at the outset. Then, it is also good to highlight the integration of gender into primary healthcare practices.

- Objectives:

- The objective of the study should be formulated by specifically mentioning the lessons learned in the integration of gender into the primary healthcare system of Ethiopia.

- Methods:

- What is the theoretical foundation (model) basis for the gender integration in the primary healthcare system of Ethiopia? Is there any use of the well-known gender integration models?

[Note: HTML markup is below. Please do not edit.]

Reviewers' comments:

Reviewer's Responses to Questions

**Comments to the Author**

1. Does this manuscript meet PLOS Global Public Health’s publication criteria? Is the manuscript technically sound, and do the data support the conclusions? The manuscript must describe methodologically and ethically rigorous research with conclusions that are appropriately drawn based on the data presented.

Reviewer #1: Yes

Reviewer #2: Yes

**********

2. Has the statistical analysis been performed appropriately and rigorously?

Reviewer #1: Yes

Reviewer #2: Yes

**********

3. Have the authors made all data underlying the findings in their manuscript fully available (please refer to the Data Availability Statement at the start of the manuscript PDF file)?

The PLOS Data policy requires authors to make all data underlying the findings described in their manuscript fully available without restriction, with rare exception. The data should be provided as part of the manuscript or its supporting information, or deposited to a public repository. For example, in addition to summary statistics, the data points behind means, medians and variance measures should be available. If there are restrictions on publicly sharing data—e.g. participant privacy or use of data from a third party—those must be specified.

Reviewer #1: Yes

Reviewer #2: Yes

**********

4. Is the manuscript presented in an intelligible fashion and written in standard English?

PLOS Global Public Health does not copyedit accepted manuscripts, so the language in submitted articles must be clear, correct, and unambiguous. Any typographical or grammatical errors should be corrected at revision, so please note any specific errors here.

Reviewer #1: Yes

Reviewer #2: Yes

**********

5. Review Comments to the Author

Please use the space provided to explain your answers to the questions above. You may also include additional comments for the author, including concerns about dual publication, research ethics, or publication ethics. (Please upload your review as an attachment if it exceeds 20,000 characters)

Reviewer #1: PLOS Reviewer’s Comments: PGPH-D-26-01529

05/27/2026

This study, Advancing Gender Equity in Primary Health Care: Lessons from Integrating Gender-Responsive Strategies into Service Delivery in Ethiopia, describes an impressive initiative to improve gender equity in maternal and child health services and outcomes.

This study was conducted by a robust team of authors with deep roots in the field. They are familiar with Ethiopian health system priorities and designed their study and interventions to accelerate gender equity.

The robust study design included “systematic, evidence-based quasi-experimental embedded implementation research with participatory multi-method approaches to integrate gender considerations into PHC. The process included before-and-after controlled household studies, phenomenological research, gender analysis, and stakeholder engagement to identify barriers and opportunities in addressing gender disparities in health care access.”

Findings from participant surveys, qualitative interviews and focus groups guided gender-intentional strategies to improve health service delivery. Research and interventions were tied to the Improve Primary Health Care Service Delivery (IPHCSD) project to strengthen the country’s PHC system and enhance RMNCH outcomes. These interventions, linked with evaluation, monitoring and accountability, have the potential to transform the Ethiopian PHC system and to improve RMNCH.

Congratulations on this outstanding study which effectively describes the “transformative potential of gender-focused strategies in overcoming health inequities and the positive influence on accessing and delivering RMNCH services at the PHC level.”

Following are suggestions for minor edits and/or improvements; numbers indicate lines in the text:

64: To set the stage, provide maternal and infant mortality statistics for Ethiopia including regional variations.

79: “the systematic integration of gender considerations into RMNCH programs remains limited” in the discussion, revisit this statement with suggestions for how these findings might be applied and extended nation-wide.

86: Change identifies to identified to make the verbs consistent.

94: Reverse the order of primary care system components from “primary level includes primary hospitals, health centers, and health posts” to the opposite. Most conventions describe PHC as starting from the lowest level of health posts; in some contexts, hospitals are considered as secondary levels of care.

139: It would be helpful to provide a summary of empathic formative research findings in an appendix.

147: Elaborate on the human-centered design approaches, either in the introduction or later in the discussion.

175: Describe or display key elements of the action plan.

196: Change strengthened to strengthen.

211-232: Gender-related barriers and strategies are critical to the study. Well done!

269: Bravo for recognizing the crucial importance of community participation and for outreach to vulnerable populations.

292: Provide examples of gender-sensitive indicators and tools; could be in appendix or see next comment.

305: Provide references or access to resources on request.

332: Figure 4 is powerful and could be used in trainings throughout the health system.

368: Why is there such a big difference between institutional delivery rates between the intervention and comparison groups? The reported rates are confusing.

403: Address how gender-intentional, community-based delivery of MNH services by community volunteers can be expanded and sustained.

414: Discuss barriers to male engagement and how this study addressed them.

434: Suggest strategies to expand and further study the outcomes of these interventions.

Reviewer #2: Introduction: The section will benefit by defining gender integration paradigms at the outset. Then, it is also good to highlight the integration of gender into primary healthcare practices.

Objectives: The objective of the study should be formulated by specifically mentioning the lessons learned in the integration of gender into the primary healthcare system of Ethiopia

Methods: What is the theoretical foundation (model) basis for the gender integration in the primary healthcare system of Ethiopia? Is there any use of the well-known gender integration models?

Results: well written

Discussion: Well written

Conclusion: Well written

**********

6. PLOS authors have the option to publish the peer review history of their article (what does this mean?). If published, this will include your full peer review and any attached files.

**Do you want your identity to be public for this peer review?**  If you choose “no”, your identity will remain anonymous but your review may still be made public.

For information about this choice, including consent withdrawal, please see our Privacy Policy.

Reviewer #1: **Yes:**  Cynthia Haq

Reviewer #2: **Yes:**  Wubegzier Mekonnen

**********

[NOTE: If reviewer comments were submitted as an attachment file, they will be attached to this email and accessible via the submission site. Please log into your account, locate the manuscript record, and check for the action link "View Attachments". If this link does not appear, there are no attachment files.]

Figure Resubmissions:

While revising your submission, we strongly recommend that you use PLOS’s NAAS tool (https://ngplosjournals.pagemajik.ai/artanalysis) to test your figure files. NAAS can convert your figure files to the TIFF file type and meet basic requirements (such as print size, resolution), or provide you with a report on issues that do not meet our requirements and that NAAS cannot fix.

After uploading your figures to PLOS’s NAAS tool - https://ngplosjournals.pagemajik.ai/artanalysis, NAAS will process the files provided and display the results in the "Uploaded Files" section of the page as the processing is complete. If the uploaded figures meet our requirements (or NAAS is able to fix the files to meet our requirements), the figure will be marked as "fixed" above. If NAAS is unable to fix the files, a red "failed" label will appear above. When NAAS has confirmed that the figure files meet our requirements, please download the file via the download option, and include these NAAS processed figure files when submitting your revised manuscript.

Attachment Submitted filename: PGPH-D-26-01529.review.ch.docx

# Author comment: Author response to Decision Letter 1

**DOI:** 10.1371/journal.pgph.0006764.r002

12 Jun 2026

Attachment Submitted filename: Point-by-point response to editor and reviewers.docx

# Editor report: Decision Letter 1

**DOI:** 10.1371/journal.pgph.0006764.r003

**Authors:** Damen Haile Mariam

28 Jun 2026

Advancing Gender Equity in Primary Health Care: Lessons from Integrating Gender-Responsive Strategies into Service Delivery in Ethiopia

PGPH-D-26-01529R1

Dear Dr. Tiruneh,

We are pleased to inform you that your manuscript 'Advancing Gender Equity in Primary Health Care: Lessons from Integrating Gender-Responsive Strategies into Service Delivery in Ethiopia' has been provisionally accepted for publication in PLOS Global Public Health.

Before your manuscript can be formally accepted you will need to complete some formatting changes, which you will receive in a follow up email. A member of our team will be in touch with a set of requests.

Please note that your manuscript will not be scheduled for publication until you have made the required changes, so a swift response is appreciated.

IMPORTANT: The editorial review process is now complete. PLOS will only permit corrections to spelling, formatting or significant scientific errors from this point onwards. Requests for major changes, or any which affect the scientific understanding of your work, will cause delays to the publication date of your manuscript.

If your institution or institutions have a press office, please notify them about your upcoming paper to help maximize its impact. If they'll be preparing press materials, please inform our press team as soon as possible -- no later than 48 hours after receiving the formal acceptance. Your manuscript will remain under strict press embargo until 2 pm Eastern Time on the date of publication. For more information, please contact globalpubhealth@plos.org.

Thank you again for supporting Open Access publishing; we are looking forward to publishing your work in PLOS Global Public Health.

Best regards,

Damen Haile Mariam, MD, MPH, PhD

Academic Editor

PLOS Global Public Health

***********************************************************

Reviewer Comments (if any, and for reference):
